# Effects of taurine, brimonidine and betaxolol on oscillation modulation and stimulation efficiency in degenerated *rd10* mouse retinas

**DOI:** 10.1038/s41598-025-06440-9

**Published:** 2025-06-20

**Authors:** Kim Schaffrath, Claudia Ingensiep, Frank Müller, Peter Walter, Sandra Johnen

**Affiliations:** 1 Department of Ophthalmology, Uniklinik RWTH Aachen, Pauwelsstraße 30, 52074 Aachen, Germany; 2 Institute of Biological Information Processing, Molecular and Cellular Physiology (IBI-1), Forschungszentrum Jülich GmbH, Wilhelm-Johnen-Straße, 52428 Jülich, Germany

**Keywords:** Retinitis pigmentosa, *rd10* mouse model, Multielectrode arrays, Neuroprotective substances, Hereditary eye disease, Retinal diseases, Vision disorders

## Abstract

The *rd10* mouse is a widely used model for degenerative retinal diseases such as retinitis pigmentosa (RP). Its retina shows rhythmic spontaneous activity at a frequency of three to seven Hz, and the retinal ganglion cells (RGCs) are less electrically excitable. We hypothesize that the electrical excitability can be improved by suppressing the oscillations using the neuroprotective drugs 2-aminoethanesulphonic acid (taurine), brimonidine and betaxolol. These are involved in calcium homeostasis and may play a crucial role in neuroprotection and excitotoxicity by preventing Ca^2+^ overload. Spontaneous activity and responses to electrical stimulation of isolated retinas from 3- to 4-month-old *rd10* mice were recorded using multielectrode arrays. At defined times, the neuroprotectants were repeatedly added to the medium according to a standardized protocol to analyze the reproducibility and reversibility of their effects. Taurine and betaxolol significantly reduced oscillations and bursting behavior and ameliorated electrical efficiency. Brimonidine only reduced the frequency of oscillations. The effects on oscillation, spontaneous firing frequency, bursting behavior and stimulation efficiency were reproducible and reversible. The drugs tested appear to be promising therapeutic candidates for improving the residual function of RGCs. They will be further investigated and combined with other RP treatments, such as retinal prostheses, in the future.

**Supplementary Information:**

The online version contains supplementary material available at 10.1038/s41598-025-06440-9.

## Introduction

Retinal dystrophies and degenerations are progressive diseases characterized by the loss of retinal cells, mainly photoreceptors and retinal pigment epithelial (RPE) cells. They are caused either by a genetic defect or, with increasing age, by environmental and genetic factors. Examples include retinitis pigmentosa (RP) and geographic atrophy (GA), one of the two advanced forms of age-related macular degeneration (AMD).

In RP, the most common inherited retinal dystrophy with a prevalence of approximately 1:4000^1,2^, rod photoreceptors degenerate first, followed by cone degeneration, leading to night blindness, peripheral visual loss, and ultimately advanced vision loss to blindness. The disease is heterogeneous, and mutations have been identified in more than 110 different genes (RetNet, https://retnet.org/, accessed March 6, 2025), inherited either as an autosomal dominant, autosomal recessive or X-linked trait. Due to its genetic heterogeneity, treatment of RP remains challenging and is not the same for every patient. It includes symptomatic therapies (e.g., visual aids)^3–5^, retinal prostheses^6^, and gene therapy for specific mutations (e.g., voretigene neparvovec for the treatment of bi-allelic mutations in the *RPE65* gene)^7^.

AMD, the leading cause of irreversible blindness in people over the age of 60 in developed countries, is expected to affect 288 million people worldwide by 2040^8^. It can progress to two advanced forms: atrophic AMD and exudative AMD, the latter affecting only 10–15% of patients. While anti-VEGF therapy has revolutionized the treatment of exudative AMD^9^, two therapeutics for end stage of dry AMD (GA) have only recently become available and have been approved by the U.S. Food and Drug Administration in 2023^10,11^. However, their approval in Europe has been withdrawn^12^. The reason given was that although atrophy progression was significantly slowed, this did not lead to a clinically relevant functional benefit for patients. Another treatment approach for GA is the PRIMA prosthesis, in which photovoltaic pixels convert projected light directly into electrical current patterns. Data from a first-in-human clinical trial confirmed that implantation was feasible and well tolerated without compromising natural peripheral vision, and that the prosthesis provided reliable letter recognition^13^.

Retinal prostheses were considered a promising technology for patients with RP and GA and were commercially available, but high costs and lower than expected efficacy led to their withdrawal from the market. One reason for the low efficacy was the failure to consider the retinal remodeling that occurs because of photoreceptor degeneration. It includes neuronal death and migration, glial cell migration, formation of new neurites and synapses, rewiring of retinal circuits, glial hypertrophy and formation of a fibrotic glial seal^14–16^.

Remodeling also occurs in animal models of retinal degeneration, including the *rd10* mouse, which is characterized by a point mutation in the gene encoding the beta subunit of rod cGMP phosphodiesterase. This gene has also been identified as a causative gene in patients with RP and congenital stationary night blindness^17^, making the *rd10* mouse a good animal model for the autosomal recessive form of RP. Compared to *rd1* retinas, *rd10* retinas show a delayed onset and slower progression of degeneration, with first histological changes at postnatal day 16 (P16) and disappearance of the a- and b-waves in the electroretinogram within the first 2 months of life^18^. Explanted *rd10* retinas show additional pathological features of electrical activity, such as the occurrence of oscillations in the range of three to seven Hz and a reduced stimulation efficiency^19–21^. The origin of the oscillations is not fully understood, but photoreceptor degeneration and subsequent inner retinal remodeling appear to play an important role. One model describes the interaction between residual cone photoreceptors, rod bipolar cells and horizontal cells, where oscillations are seen as a consequence of synaptic remodeling triggered by photoreceptor death^22^. Another model suggests recurrent interactions between AII amacrine cells and cone bipolar cells, with voltage-gated Na^+^ and K^+^ channels of AII amacrine cells playing an important role as intrinsic triggers^23–25^.

Characteristics of electrical retinal activity can be measured with multielectrode arrays (MEAs), which are used to study complex neuronal networks ex vivo. MEAs consist of several metal electrodes arranged in an array that can be used to record neuronal signals or emit electrical signals. They are also used to study the effects of inhibitory or neuroprotective substances on neuronal networks.

Neuroprotective substances serve to maintain the physiological function of neurons and prevent or slow their degeneration. Mechanisms of action range from beneficial effects on mitochondrial dysfunction, attenuation or inhibition of apoptosis, excitotoxicity, inflammation, oxidative stress and protein misfolding to neurotrophic activity, modulation of neuronal survival signaling pathways, autophagy, and epigenetics^26^. Neuroprotective substances include chemical and naturally occurring molecules as well as proteins. Our literature research identified taurine, brimonidine and betaxolol as promising candidates. Taurine (2-aminoethanesulfonic acid), which, unlike other amino acids, has a sulfonic acid instead of a carboxyl group. Taurine is ubiquitous in most mammalian cells and tissues. It plays a critical role in modulating Ca^2+^ homeostasis, has antioxidant, anti-apoptotic, anti-inflammatory and neuromodulatory activities, attenuates endoplasmic reticulum stress, regulates gene expression, and serves as an organic osmolyte^27^. In the retina, taurine deficiency induces oxidative stress and apoptosis and results in degeneration of photoreceptors and retinal ganglion cells (RGCs)^28–30^.

A second molecule is brimonidine (brimonidine tartrate), which belongs to the group of sympathomimetics. As a selective alpha-2 adrenergic receptor agonist, it reduces aqueous humor production and increases uveoscleral outflow in the treatment of glaucoma or ocular hypertension^31^. In addition, brimonidine has a neuroprotective effect on retinal neurons, as demonstrated in in vivo models of elevated intraocular pressure^32,33^ and phototoxicity^34,35^. In a phase 2b clinical study, intravitreal brimonidine was shown to reduce GA growth^36^. A third molecule is betaxolol, a selective beta-1 adrenergic receptor antagonist used to treat hypertension and glaucoma. In the eye, it slows the production of aqueous humor, thereby lowering intraocular pressure^37^. Further studies have shown that betaxolol has a neuroprotective effect, probably by blocking calcium entry through voltage-gated calcium channels^38,39^.

Many studies demonstrating neuroprotective effects on RGCs are based on histological, immunohistochemical, protein biochemical, and molecular biological analyses rather than on functional investigations. The aim of our study was to investigate the effect of taurine, brimonidine and betaxolol on pathological RGC activity—the occurrence of oscillations and reduced stimulation efficiency—in *rd10* retinas ex vivo using MEAs. In contrast to previous studies, we analyzed the electrophysiological effects of these substances in degenerative retinal diseases.

## Results

### Drug-induced modulatory potential on the oscillatory activity of the *rd10* retina

**Fig. 1:**
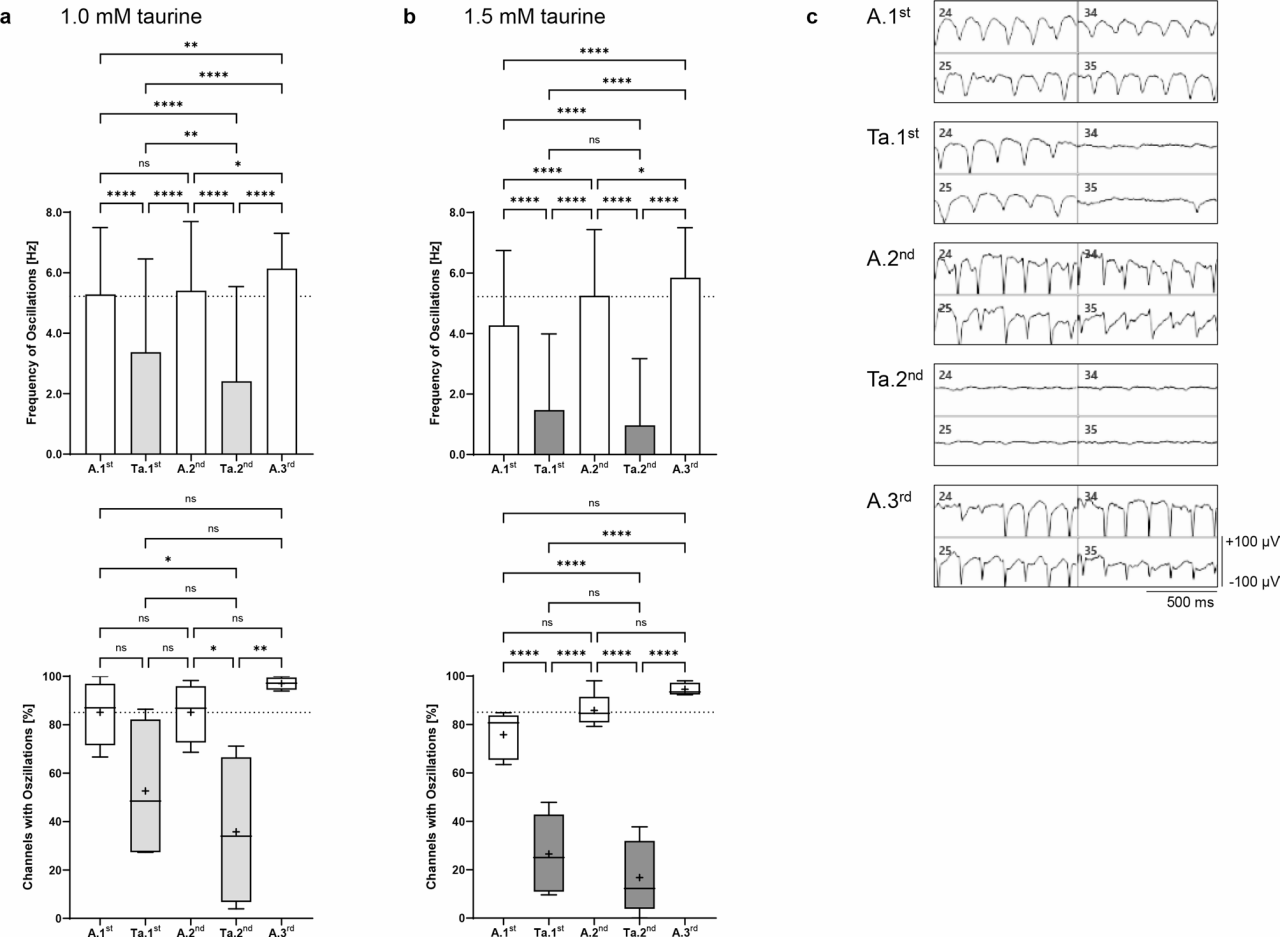
Effect of taurine on the oscillation frequency of local field potentials in *rd10* retinas. The upper graphs show the analysis of the oscillation frequencies [Hz] during the individual perfusion steps with Ames’ medium (A.1^st^–A.3^rd^, white) and (**a**) 1.0 mM taurine (Ta.1^st^–Ta.2^nd^, light grey) or (**b**) 1.5 mM taurine (dark grey). (179 channels analyzed for 1.0 mM taurine; 259–260 channels analyzed for 1.5 mM taurine). The lower graphs show the number of active channels with oscillations [%] during perfusion with Ames’ medium and 1.0 mM or 1.5 mM taurine. Data are presented as box-and-whisker plots (33–59 channels included for 1.0 mM taurine; 46–57 channels included for 1.5 mM taurine). The mean values of the oscillation frequency and the percentage recording channels with oscillations during the first Ames’ perfusion from all experiments are shown as dotted lines. (**c**) Representative recordings of four selected MEA channels during alternating perfusion with Ames’ medium and 1.5 mM taurine. The 50 Hz low pass filtered data illustrate the repetitive appearance and disappearance of oscillations (x-axis scale: 500 ms, y-axis scale: ± 100 µV).

**Fig. 2:**
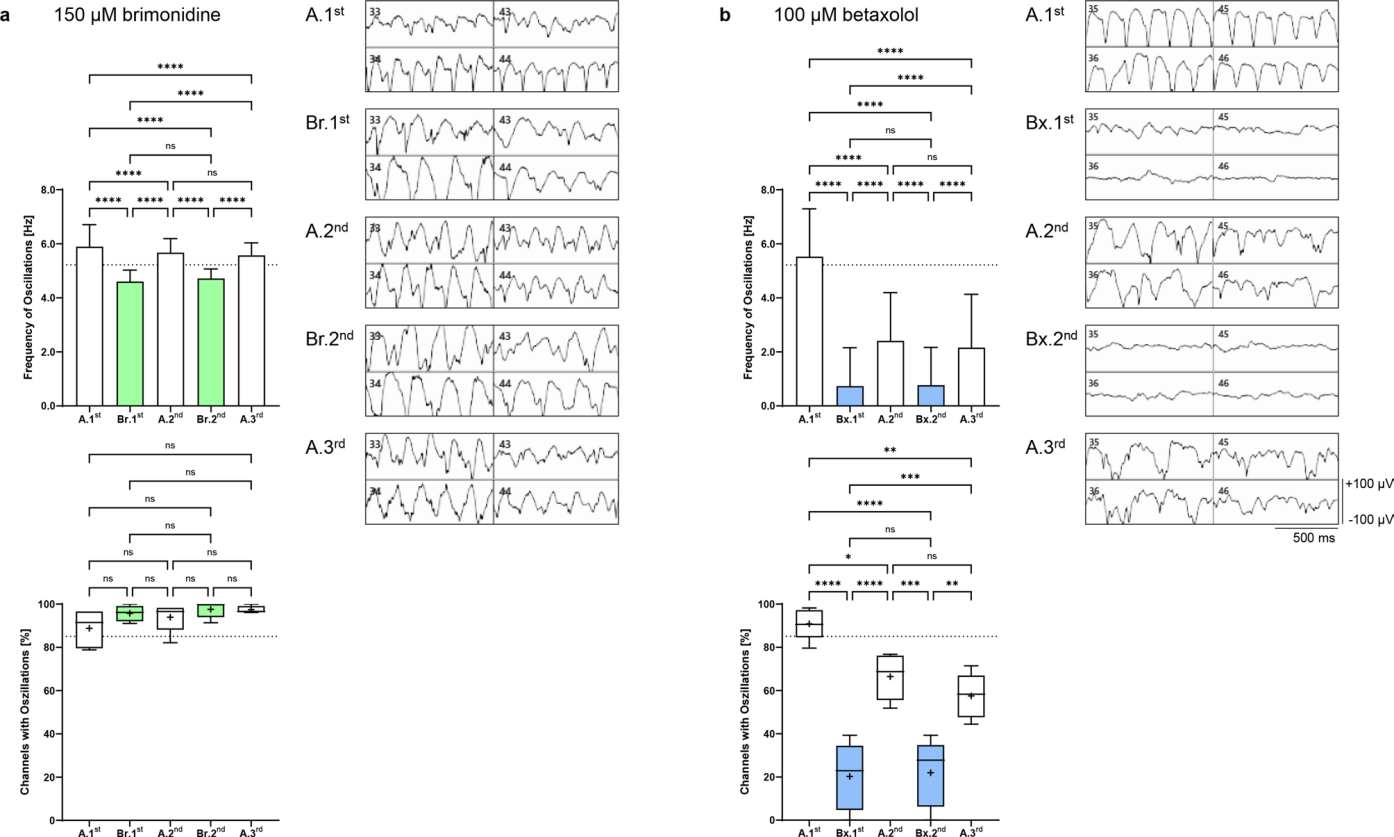
Effect of brimonidine and betaxolol on the oscillation frequency of local field potentials in *rd10* retinas. The upper graphs show the analysis of the oscillation frequencies [Hz] during the individual perfusion steps with Ames’ medium (A.1^st^–A.3^rd^, white) and (**a**) 150 µM brimonidine (Br.1^st^–Br.2^nd^, light green) or (**b**) 100 µM betaxolol (Bx.1^st^–Bx.2^nd^, light blue). (247–277 channels analyzed for 150 µM brimonidine; 243 channels analyzed for 100 µM betaxolol). The lower graphs show the number of active channels with oscillations [%] during perfusion with Ames’ medium and 150 µM brimonidine or 100 µM betaxolol. Data are presented as box-and-whisker plots (not significant, 52–59 channels included for 150 µM brimonidine, 32–56 channels included for 100 µM betaxolol). The mean values of the oscillation frequency and the percentage recording channels with oscillations during the first Ames’ perfusion from all experiments are shown as dotted lines. The panels show representative recordings of four selected MEA channels during alternating perfusion with Ames’ medium and (**a**) 150 µM brimonidine or (**b**) 100 µM betaxolol. The 50 Hz low-pass filtered data illustrate the persistence of oscillations during brimonidine wash-in and the repeated disappearance of oscillations during betaxolol wash-in (x-axis scale: 500 ms, y-axis scale: ± 100 µV).

As many other studies have previously shown, reproducible oscillations with robust frequencies of three to seven Hz occur in local field potentials (LFPs) in 3- to 4-month-old *rd10* mice^19,21,40^. Summarizing all MEA experiments, we observed oscillations with an initial mean value of 5.22 ± 2.01 Hz measured in 85.1 ± 10.9% of the recording channels during the first Ames’ perfusion (A.1^st^; shown as baselines in Figs. 1 and 2).

With the addition of 1.0 mM taurine, the frequency of *rd10* oscillations decreased to 3.37 ± 3.09 Hz during the first wash-in and to 2.41 ± 3.13 Hz during the second wash-in (*p* < 0.0001). Oscillations were detected in only 52.7 ± 30.0% (*p* = 0.24) and 35.8 ± 31.5% (*p* = 0.03) of the recording channels, respectively, compared to 85.2 ± 13.8% during the initial perfusion with Ames’ medium (Fig. 1a). Increasing the taurine concentration to 1.5 mM resulted in a further decrease of oscillations to a frequency of 1.47 ± 2.52 Hz (56.4% less) and 0.97 ± 2.20 Hz (59.8% less) (*p* < 0.0001, Ta.1^st^ vs. Ta.2^nd^: not significant), with a significant dose-dependent decrease between 1.0 mM and 1.5 mM taurine (two-tailed t-test, Ta.1^st^ and Ta.2^nd^, *p* < 0.0001) and a reduced number of electrodes with oscillations (from initially 75.8 ± 9.72% to 26.5 ± 16.4% and 16.8 ± 15.1%, *p* < 0.0001; Fig. 1b) without a dose-dependent difference (two-tailed t-test with 1.0 mM and 1.5 mM taurine, Ta.1^st^ and Ta.2^nd^, not significant). Based on the perfusion protocol used, a significantly reversible and reproducible effect of both 1.0 mM and 1.5 mM taurine was observed. Oscillation frequency was significantly reduced, and oscillations were even abolished in many electrodes (Fig. 1c).

During the first wash-in of 150 µM brimonidine, all channels showed oscillations with a frequency of 4.60 ± 0.42 Hz, which was reduced by only 21.9% compared to the initial frequency of 5.89 ± 0.81 Hz (*p* < 0.0001). The second wash-in showed the same reversible and reproducible effect (4.71 ± 0.35 Hz, corresponding to a reduction of 20.0% compared to the initial value, *p* < 0.0001, Br.1^st^ vs. Br.2^nd^: not significant). The number of channels with oscillations ranged from 88.8 ± 8.64% to 97.6 ± 3.77% throughout the experiment (not significant, Fig. 2a).

The addition of 100 µM betaxolol to the perfusion medium resulted in a significant reduction of the oscillation frequency from an initial 5.52 ± 1.77 Hz to 0.74 ± 1.42 Hz and 0.77 ± 1.40 Hz (reduction of approximately 86%, *p* < 0.0001, Bx.1^st^ vs. Bx.2^nd^: not significant). However, after the second and third perfusion with Ames’ medium, the oscillation frequency did not return to the initial value but decreased by 56.3% and 60.9% to 2.41 ± 1.79 Hz and 2.16 ± 1.98 Hz, respectively. The number of channels with oscillations decreased from 90.9 ± 7.26% to 66.4 ± 10.7% and 57.5 ± 10.4% during the Ames’ medium perfusions (A.^1st^ vs. A.^2nd^: *p* = 0.03, A.^1st^ vs. A.^3rd^: *p* = 0.003, A.^2nd^ vs. A.^3rd^: not significant) and was only 20.3 ± 15.7% to 22.0 ± 15.6% during the two betaxolol perfusions (*p* < 0.0001, A.^2nd^ vs Bx.2^nd^: *p* = 0.0001; Fig. 2b). We observed a partially reversible and fully reproducible effect with betaxolol (Bx.1^st^ vs. Bx.2^nd^: not significant).

### Effect of the drugs on the spontaneous firing frequency of the *rd10* retina

Spontaneous firing frequency was similar in all initial perfusions with Ames’ medium and ranged from 14.1 ± 15.0 Hz to 22.1 ± 15.9 Hz at cell level and from 33.6 ± 50.1 Hz to 52.6 ± 52.8 Hz at channel level. For all drugs except for brimonidine, the effect on spontaneous firing frequency was reversible and reproducible.

We performed the analysis of spontaneous firing frequency at both cell and channel level because we wanted to analyze whether there was a visible difference between cell and electrode/channel level. Due to spike sorting, many spikes were excluded from the cell-level analysis. We observed a higher firing rate at channel level.

**Fig. 3:**
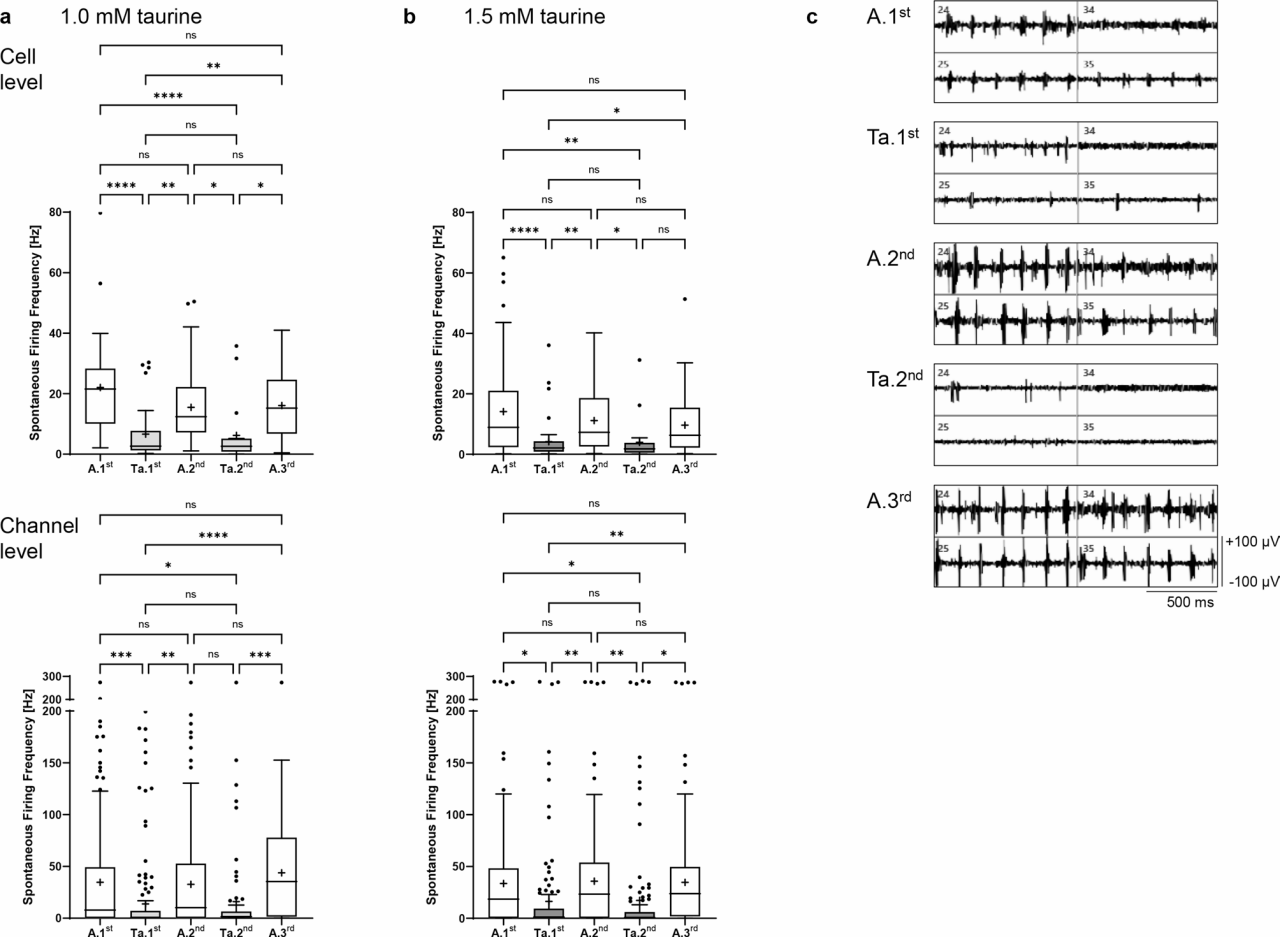
Effect of taurine on the spontaneous firing frequency of *rd10* neurons. Analysis of the spontaneous firing frequency [Hz] at cell level (upper graphs) and at channel level (lower graphs) during the individual perfusion steps with Ames’ medium (A.1^st^–A.3^rd^, white) and (**a**) 1.0 mM taurine (Ta.1^st^–Ta.2^nd^, light grey) or (**b**) 1.5 mM taurine (dark grey). Data are presented as box-and-whisker plots (18–70 cells analyzed for 1.0 mM taurine; 21–99 cells analyzed for 1.5 mM taurine; 70–178 channels analyzed for 1.0 mM taurine; 138–185 channels analyzed for 1.5 mM taurine). (**c**) Representative recordings of four selected MEA channels during alternating perfusion with Ames’ medium and 1.5 mM taurine. The 200–2000 Hz band-pass filtered data illustrate the spontaneous firing activity of the differently perfused *rd10* retina (x-axis scale: 500 ms, y-axis scale: ± 100 µV).

With 1 mM taurine, the spontaneous firing frequency at cell level decreased by 69.9% to 6.63 ± 8.77 Hz during the first wash-in (*p* < 0.0001), increased to 15.47 ± 11.31 Hz during the second Ames’ perfusion (*p* = 0.002), decreased by 60.0% to 6.20 ± 10.51 Hz during the second wash-in (*p* = 0.023), and increased again to a final frequency of 16.1 ± 11.0 Hz during the third Ames’ perfusion step (*p* = 0.015). At channel level, the first wash-in resulted in a 2.5-fold reduction in spontaneous firing frequency (from 34.7 ± 52.5 Hz to 13.8 ± 37.6 Hz, *p* = 0.0005) and the second wash-in resulted in a 2.0-fold reduction (from 32.7 ± 46.6 Hz to 16.0 ± 43.5 Hz, *p* = 0.071; Fig. 3a). Spontaneous firing frequency was similar between all three Ames’ and between the two taurine perfusion steps (for 1.0 and 1.5 mM), respectively (not significant). Perfusion with 1.5 mM taurine had the same effects. At cell level, a decrease to 4.17 ± 6.89 Hz (first wash-in, *p* < 0.0001) and 3.98 ± 7.16 Hz (second wash-in, *p* = 0.046) was observed, which is a reduction of 70.4% and 64.5%, respectively, compared to the previous Ames’ perfusion step. At channel level, a 2.1-fold reduction (from 33.6 ± 50.1 Hz to 16.3 ± 46.3 Hz, *p* = 0.018, and from 35.8 ± 49.0 Hz to 17.3 ± 51.9 Hz, *p* = 0.007) was observed during the two wash-in steps (Fig. 3b,c).

**Fig. 4:**
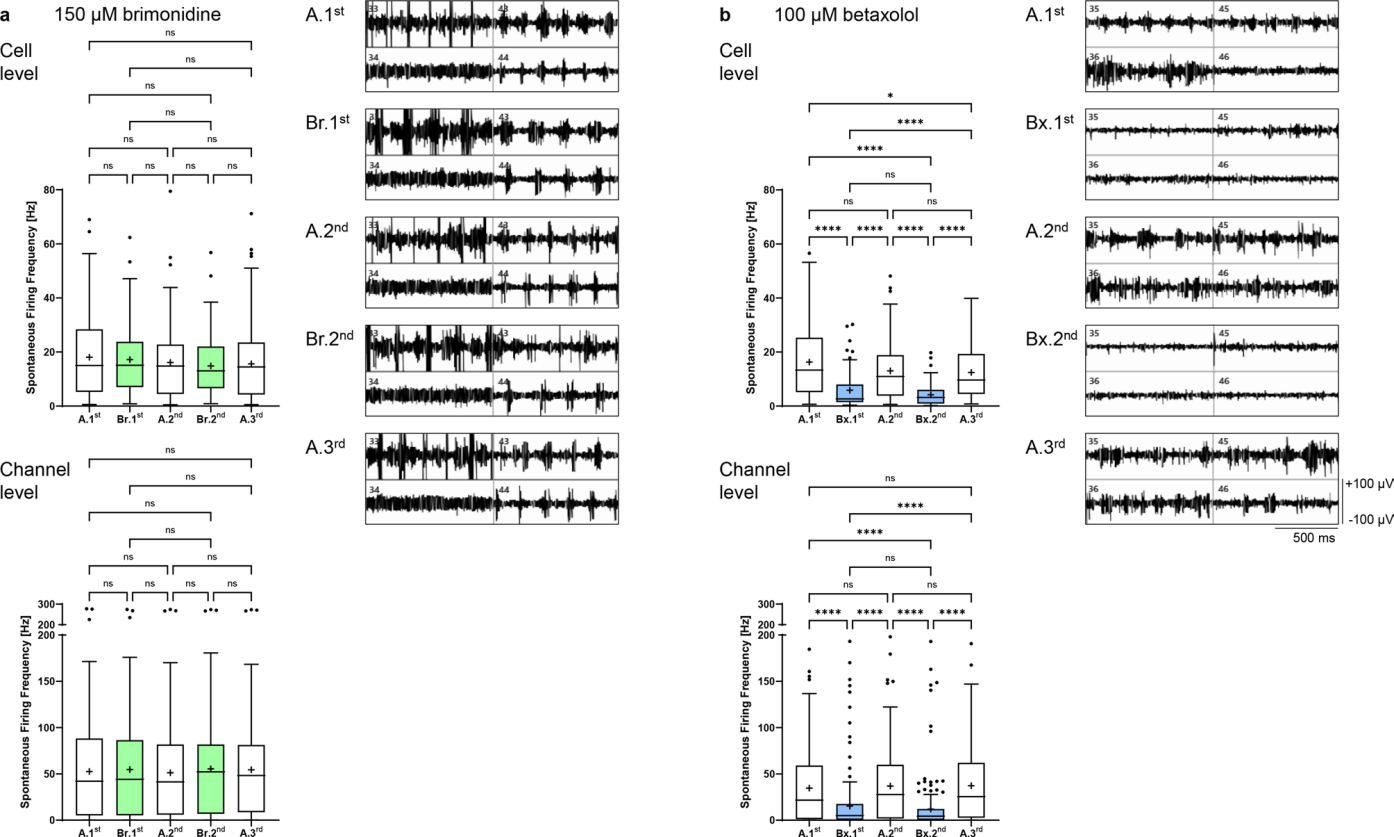
Effect of brimonidine and betaxolol on the spontaneous firing frequency of *rd10* neurons. Analysis of the spontaneous firing frequency [Hz] at cell level (upper graphs) and at channel level (lower graphs) during the individual perfusion steps with Ames’ medium (A.1^st^–A.3^rd^, white) and (**a**) 150 µM brimonidine (Br.1^st^–Br.2^nd^, light green) or (**b**) 100 µM betaxolol (Bx.1^st^–Bx.2^nd^, light blue). Data are presented as box-and-whisker plots (120–172 cells analyzed for 150 µM brimonidine; 81–156 cells analyzed for 100 µM betaxolol; 208–231 channels analyzed for 150 µM brimonidine; 212–231 channels analyzed for 100 µM betaxolol). The panels show representative recordings of four selected MEA channels during alternating perfusion with Ames’ medium and (**a**) 150 µM brimonidine or (**b**) 100 µM betaxolol. The 200–2000 Hz band-pass filtered data illustrate the spontaneous firing activity of the differently perfused *rd10* retina (x-axis scale: 500 ms, y-axis scale: ± 100 µV).

Both wash-in steps with 150 µM brimonidine had no significant effect on spontaneous firing frequency, neither at cell level (17.2 ± 12.1 Hz and 14.8 ± 10.3 Hz for Br.1^st^ and Br.2^nd^ compared to 18.1 ± 14.5 Hz, 16.1 ± 13.0 Hz and 15.6 ± 12.9 Hz for A.1^st^-A.3^rd^) nor at channel level (54.7 ± 52.5 Hz and 55.5 ± 51.0 Hz for Br.1^st^ and Br.2^nd^ compared to 52.6 ± 52.8 Hz, 51.3 ± 50.6 Hz and 54.4 ± 50.1 Hz for A.1^st^-A.3^rd^; Fig. 4a).

With 100 µM betaxolol, the spontaneous firing frequency at cell level decreased by 64.1% from 16.3 ± 12.6 Hz to 5.85 ± 6.72 Hz during the first wash-in, increased to 13.0 ± 10.7 Hz during the second Ames’ perfusion, decreased by 67.8% to 4.18 ± 4.00 Hz during the second wash-in and increased again to a frequency of 12.4 ± 9.57 Hz during the third Ames’ perfusion step (*p* < 0.0001). At channel level, the first wash-in resulted in a 2.3-fold reduction in spontaneous firing frequency (from 34.7 ± 39.1 Hz during A.1^st^ to 15.2 ± 28.7 Hz during Bx.1^st^, *p* < 0.0001); the second wash-in resulted in a 3.1-fold reduction (from 36.9 ± 37.8 Hz during A.2^nd^ to 12.1 ± 26.1 Hz during Bx.2^nd^, *p* < 0.0001; Fig. 4b).

### Effect of the drugs on the bursting behavior of the *rd10* retina

**Fig. 5:**
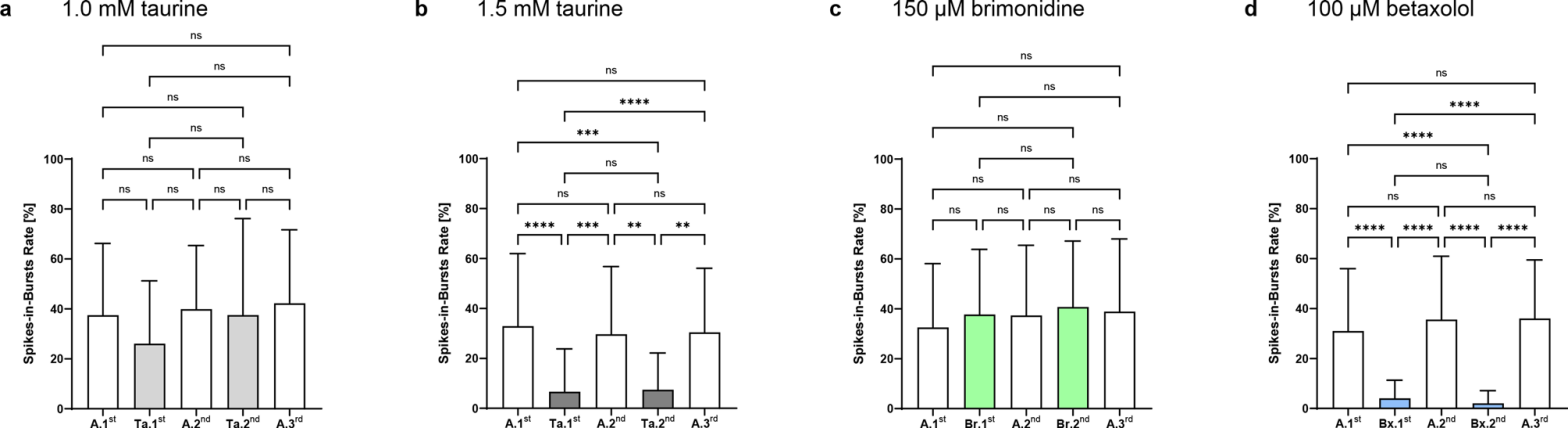
Effect of taurine, brimonidine and betaxolol on the bursting behavior of *rd10* neurons. Analysis of the number of spikes in bursts [%] during the individual perfusion steps with Ames’ medium (A.1^st^–A.3^rd^, white) and (**a**) 1.0 mM taurine (Ta.1^st^–Ta.2^nd^, light grey; 16–70 cells analyzed), (**b**) 1.5 mM taurine (Ta.1^st^–Ta.2^nd^, dark grey; 21–99 cells analyzed), (**c**) 150 µM brimonidine (Br.1^st^–Br.2^nd^, light green; 118–172 cells analyzed) or (**d**) 100 µM betaxolol (Bx.1^st^–Bx.2^nd^, light blue; 81–156 cells analyzed).

In addition to oscillations, bursts are another pathological component of spontaneous activity in the *rd10* retina. No significant changes in burst rates were observed when 1.0 mM taurine was used (Fig. 5a), which changed when the taurine concentration was increased to 1.5 mM. Here, the percentage of spikes fired in bursts decreased 4.9-fold from 32.9 ± 29.1% to 6.66 ± 17.1% (*p* < 0.0001). Wash-out of taurine returned the percentage to 29.7 ± 27.1% (*p* = 0.0001). Repeated taurine perfusion again reduced the burst rate by a factor of 4 to 7.41 ± 14.8% reproducibly (*p* = 0.004, Ta.1^st^ vs. Ta.2^nd^: not significant), which was again reversible (30.4 ± 25.7% during the third perfusion with Ames’ medium, *p* = 0.002, all Ames’ perfusion steps: not significant; Fig. 5b).

There were no significant changes in burst rates when 150 µM brimonidine was used. In fact, a slight increase in the percentage of spikes fired in bursts was observed (32.5 ± 25.5 during the first perfusion with Ames’ medium to 40.7 ± 26.4% during the second perfusion with brimonidine; Fig. 5c).

Perfusion with 100 µM betaxolol showed the greatest reduction in burst rates: a percentage of 4.04 ± 7.25% during the first wash-in and 2.03 ± 5.09% during the second wash-in represented a 7.7-fold and 15.3-fold decrease, respectively, from the initial percentage of 31.0 ± 25.0% (*p* < 0.0001, Bx.1^st^ vs. Bx.2^nd^: not significant). During the second and third perfusion with Ames’ medium, the burst rate returned to 35.6 ± 25.3% and 36.0 ± 23.4%, respectively (*p* < 0.0001, all Ames’ perfusion steps: not significant; Fig. 5d). Overall, the effect on bursting behavior with betaxolol was reversible and reproducible.

### Effect of the drugs on the stimulation efficiency of the *rd10* retina

**Fig. 6:**
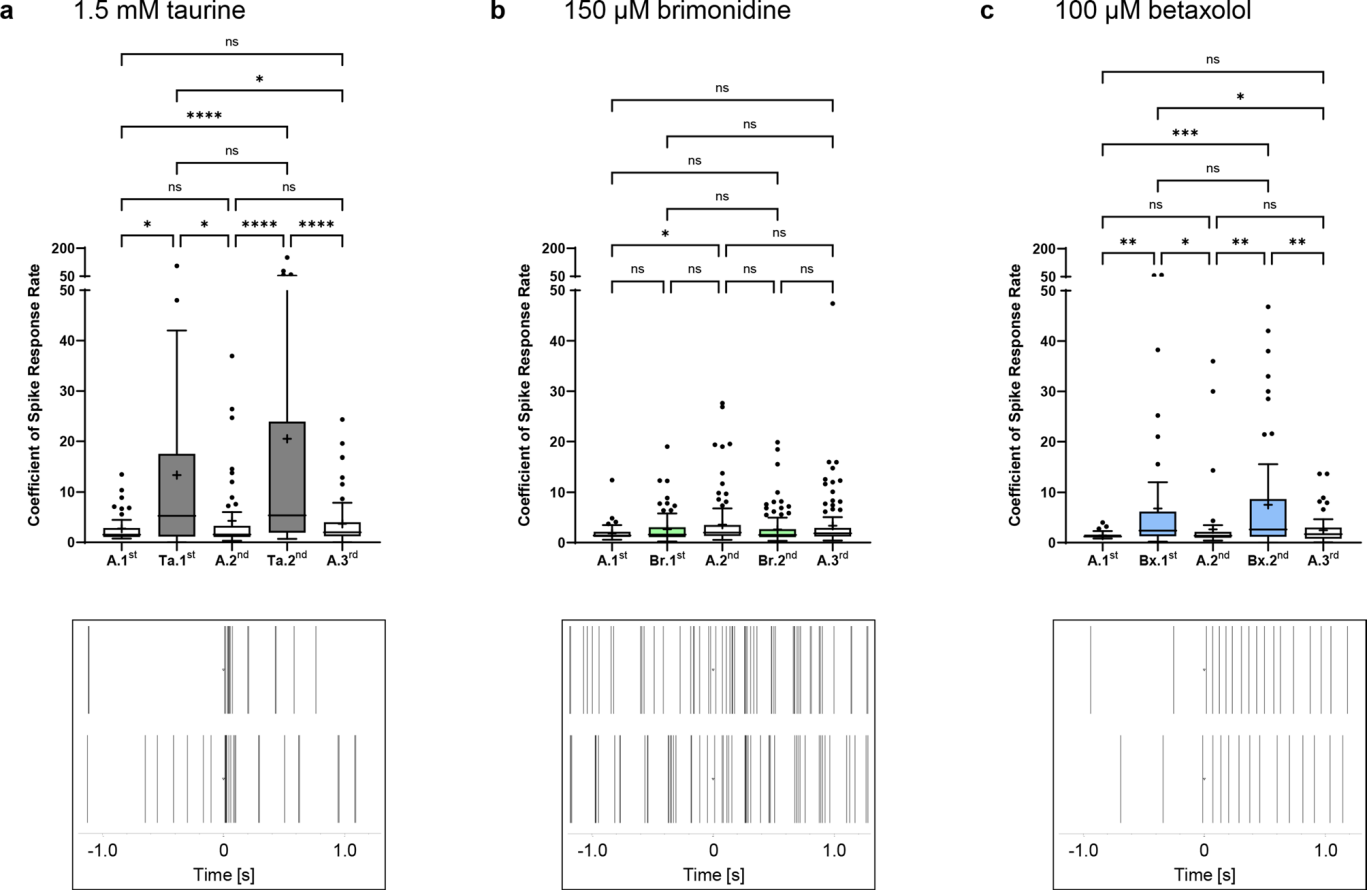
Effect of taurine, brimonidine and betaxolol on the stimulation efficiency of *rd10* neurons to electrical stimulation. The graphs show the analysis of the stimulation efficiency of *rd10* neurons to electrical stimulation (± 80 µA, 500 µs per phase) during the individual perfusion steps with Ames’ medium (A.1^st^–A.3^rd^, white) and (**a**) 1.5 mM taurine (Ta.1^st^–Ta.2^nd^, dark grey), (**b**) 150 µM brimonidine (Br.1^st^–Br.2^nd^, light green) or (**c**) 100 µM betaxolol (Bx.1^st^–Bx.2^nd^, light blue). Data are presented as box-and-whisker plots (28–61 cells analyzed for 1.5 mM taurine; 79–117 cells analyzed for 150 µM brimonidine; 50–75 cells analyzed for 100 µM betaxolol). The panels show representative recordings of individual retinal neurons (two per drug) before and after application of an electrical stimulus (± 80 µA; inverted triangle). The timestamps illustrate the sorted spikes immediately one second before and after the stimulus.

Electrical stimulation in the presence and absence of the different drugs was performed with different biphasic pulses in the range of ± 40–100 µA, with a current strength of ± 80 µA being the most effective (Fig. 6). The stimulation efficiency was calculated as the coefficient of the firing rate 3 s before the stimulus and the firing rate 0.5 s after the stimulus (spike response coefficient). During the first perfusion with 1.5 mM taurine, the spike response coefficient increased 4.9-fold from 2.73 ± 2.82 to 13.4 ± 20.1 (*p* = 0.012). The effect was reversible and could be repeated during the second taurine perfusion (4.8-fold increase from 4.27 ± 7.02 to 20.5 ± 33.5, *p* < 0.0001). With the final wash-out with Ames’ medium, the stimulation efficiency decreased again to 3.68 ± 4.67 (*p* < 0.0001; Fig. 6a). For the other current strengths tested, the maximum spike response coefficient during taurine perfusion was 14.2 ± 27.7 at ± 40 µA, 19.5 ± 36.7 at ± 60 µA and 21.1 ± 28.9 at ± 100 µA (Supplementary Fig. S1).

Perfusion with 150 µM brimonidine did not significantly increase the stimulation efficiency after stimulation with ± 80 µA. The coefficients of 2.63 ± 2.81 (Br.1^st^) and 2.62 ± 3.15 (Br.2^nd^) were even lower than those calculated for the second (3.58 ± 4.79) and third (3.36 ± 5.23) perfusion with Ames’ medium (not significant; Fig. 6b). Stimulation with ± 40 µA, ± 60 µA and ± 100 µA gave comparable results (Supplementary Fig. S2).

The use of 100 µM betaxolol resulted in a higher stimulation efficiency. When stimulated at ± 80 µA, starting from a coefficient of 1.42 ± 0.60, the first betaxolol wash-in showed a 4.8-fold increase to 6.78 ± 11.9 (*p* = 0.003), which decreased again to 2.65 ± 5.47 under Ames’ perfusion (*p* = 0.02), increased again to 7.53 ± 11.2 at the second betaxolol wash-in (*p* = 0.003), and finally decreased to 2.46 ± 2.60 (*p* = 0.002) (Fig. 6c). Betaxolol induced a reversible and reproducible effect on the stimulation efficiency (Bx.1^st^ vs. Bx.2^nd^: not significant, all Ames’ perfusion steps: not significant). The maximum spike response coefficients at ± 40 µA, ± 60 µA and ± 100 µA were 6.27 ± 20.2, 6.36 ± 19.9 and 9.16 ± 26.6, respectively (Supplementary Fig. S3).

## Discussion

Several studies have shown that taurine, brimonidine, and betaxolol have positive neuromodulatory or even neuroprotective effects on retinal thickness, photoreceptors and the ganglion cell layer in animal models of glaucoma, retinal ischemia, RP or AMD. Examples include taurine-mediated protection of RGCs in a model of episcleral vein cauterization or after photoreceptor loss in P23H rats^41^, taurine-mediated rescue of cone populations and partial maintenance of ERG function in a model of MNU-induced photoreceptor degeneration^28^, brimonidine-mediated preservation of retinal thickness and number of RGCs in a model of NMDA-induced RGC death^42^, and betaxolol-mediated reduction of photoreceptor loss and associated protection of ERG function in a model of photic-induced retinopathy^39^. Here, protection of retinal function referred to protection of the photoreceptors and maintenance of the ERG. However, to our knowledge, the effects of these substances on altered RGC function in a RP model—in terms of oscillations, spontaneous firing frequency, bursting behavior, and stimulation efficiency—have not yet been investigated. Therefore, we analyzed the modulation of RGC functionality by the above-mentioned neuroprotective drugs in retinas of 3- to 4-month-old *rd10* mice using MEA recordings in terms of the spontaneous behavior of the degenerated retinas and the effects after stimulation of the degenerated retinas. To assess the reproducibility and reversibility of the drug effects, an experimental protocol was established with two drug perfusion steps separated by a pre- and post-perfusion with Ames’ medium. Overall, the electrical activity during the first Ames’ perfusion was often lower than during the second or third Ames’ perfusion, which could be due to the stress of the preparation, although a lead time of at least 40 min was used to ensure a steady state of retinal activity.

The altered activity of the RGCs, manifested as reduced electrical excitability, suggests that the oscillations are partially responsible for the reduced effectiveness of visual prostheses in RP patients^20,21,43^. The oscillations in the LFPs as part of the aberrant activity of the RGCs result in a reduced signal-to-noise ratio. They might act as background noise that reduces the efficiency of stimulation and impairs the clarity of visual signaling in the retina and the signal transmission within the neuronal network from the eye to the brain^20,21,43–46^. The first question that arose was to examine the altered spontaneous activity and manipulate the oscillations to optimize retinal function. Here, taurine and brimonidine proved to be promising neuroprotective drugs that significantly reduced these oscillations, whereas betaxolol almost completely abolished them. At the same time, we observed a slight change in the shape of the oscillations in the LFP with betaxolol, which was not seen with taurine and brimonidine. As the origin of the oscillations is not yet fully understood, our results suggest that betaxolol interferes with the signaling pathway in a different way to taurine and brimonidine.

Reduced spontaneous firing activity and vanishing oscillations have been described for several substances in retinal degeneration models, including ionotropic glutamate receptor blockers, CNQX (an AMPA receptor antagonist), glycine, gamma-aminobutyric acid (GABA, the main inhibitory neurotransmitter in the central nervous system), and benzodiazepines^19,21,47^. In addition, various gap junction blockers (meclofenamic acid (MFA), 18-β-glycyrrhetinic acid) or flupirtine (a potassium channel opener) also showed a reduction in spontaneous firing rate and oscillations. Except for MFA, these substances reduced the sensitivity to light stimuli but improved the signal-to-noise ratio of light responses^48^. Furthermore, the suppression of oscillations, for example, induced by gap junction blockers or benzodiazepines, could induce a metabolic inhibition, reduce background noise and enhance responses to stimulation resulting in an increased stimulation efficiency^21,45,46^. Taurine’s mode of action may be explained by its structural similarity to GABA and the resulting activation of different types of GABA-as well as glycine receptors^49,50^. Brimonidine has been shown to enhance GABAergic transmission via GABA_A_ receptors, resulting in GABAergic responses^51^. Betaxolol has been shown to reduce glutamate-induced spontaneous firing rate in RGCs by reducing voltage-dependent sodium and calcium currents, thus counteracting glutamate as a GABA antagonist^52^.

In healthy retinas, the transmission of visual information from the retina to the lateral geniculate nucleus (LGN) is improved and augmented, and synaptic plasticity can be induced by bursts^53,54^. In degenerated *rd1* and *rd10* retinas, a correlation between the occurrence of bursts, the presence of oscillations and a reduced stimulation efficiency has already been investigated and showed similar results to our study^20,43,45^. Several gap junction-blockers, flupirtine and the neurotransmitters glycine, GABA, and benzodiazepines increased the efficiency of electrical stimulation while suppressing oscillations in the *rd10* retina^21,45,48^. In our experiments, increased stimulation efficiency was also observed during taurine and betaxolol perfusion, with fewer bursts and reduced or almost abolished oscillations. Previous studies by our group showed that taurine increased RGC responses and retinal cell survival under hypoxic stress conditions and counteracted increased bursting behavior under pressure conditions^55,56^.

In the literature, hyperactivity of *rd1* and *rd10* RGCs compared to wildtype RGCs was described and resulted in a reduced signal-to-noise ratio and stimulations efficiency^57^. We detected increased bursting behavior with the aberrant rhythmic activity, which could be interpreted as hyperactivity, but we did not see an increased spontaneous spiking frequency.

Despite the valuable findings of this study regarding the beneficial effects of taurine, brimonidine and betaxolol on the pathological functionality of *rd10* RGCs, some limitations must be mentioned. The drugs were tested ex vivo using whole retinas, which is an important model between in vitro and in vivo. This model is closer to the in vivo situation, as cytoarchitecture, intercellular connections and interactions are maintained; however, it does not consider systemic effects such as blood flow, immune responses and drug metabolism. Furthermore, the experiments were performed at room temperature (RT), which may lead to biased results, as temperature can affect cellular processes and drug efficacy^58,59^. Therefore, direct transferability to in vivo conditions is limited. The data also lacks a link to visual function and behavioral improvement. It is important to mention that meaningful ex vivo experiments are an important step towards in vivo studies, as they are ethically advantageous in that they do not require postoperative treatment. We demonstrated that taurine, brimonidine, and betaxolol positively affect the activity of *rd10* RGCs ex vivo. This provides the necessary basis for applying for approval of an animal experiment project and subsequent in vivo studies, in which molecular, cellular and functional issues, as well as the influence of the drugs on different stages of degeneration, can be investigated. As methodological limitation of our study we must mention that mandatory spike sorting led to loss of data if cells could not be clearly distinguished. To overcome this issue, spontaneous RGC activity was analyzed at both the cell and channel level, as shown in other studies^55^. Other limitations were the different firing behavior of RGCs, the resulting variance of data after data processing and the variance in the number of active channels, spikes, and cells depending on the substance used. Thus, due to the higher overall activity during perfusion with Ames’ and brimonidine, more cells could be analyzed than with taurine and betaxolol. Experiments were performed with *rd10* mice at 3- to 4-months of age to study retinas in a fully degenerated state (after P60), but before the peak of remodeling^40,60^. Future studies should use younger *rd10* mice to further analyze the neuroprotective effects of the substances during the degeneration phase. Therefore, the substances need to be administered before or at the beginning of the degeneration process. However, in younger *rd10* mice (P30), analysis of the fluctuating oscillations is more uncertain and complex^20^. Furthermore, it is described that the electrical activity of *rd10* retinas in the degeneration phase is changing^20,43,61^.

In neurodegenerative diseases, like e.g., AMD, glaucoma, RP, diabetic retinopathy as well as in Alzheimer’s and Parkinson’s disease, cells suffer from excitotoxicity due to imbalance in Ca^2+^ homeostasis, mitochondrial dysfunction leading to oxidative stress and neuroinflammation mediated by the purinergic receptor P2X7^62^. As taurine, brimonidine, and betaxolol interact in calcium homeostasis, they can prevent Ca^2+^ overload which is one of their neuroprotective properties.

Chen and colleagues showed that modulation of G protein coupled receptors (GPCRs) attenuated retinal oxidative stress and prevented degeneration in retinal degenerative diseases, including diabetic retinopathy. Activation of adrenergic-2 receptors via the Gi signaling pathway by, e.g., brimonidine, or inhibition of adrenergic-1 receptors by, e.g., betaxolol, or of serotonin receptors via the Gs and Gq signaling pathways could counteract the decreased activity of Gi-coupled or the increased activity of Gs and Gq-coupled GPCRs and, thus, attenuate the subsequent photoreceptor death in these degenerative diseases. This leads to the conclusion that studying the interaction of GPCR pathways might be more advantageous than focusing on single molecules^63,64^.

Therapeutic approaches for inherited retinal degenerations, regardless of the specific genetic defect, are the focus of research to address not just a single genetic variant but many affected genes and thus a larger population of patients. A combination of tamsulosin, metoprolol and bromocriptine suppressed intracellular cAMP and Ca^2+^ activity via GPCR modulation^65^. In RP, degeneration starts with rod photoreceptor death leading to hyperoxia, oxidative stress and increased intracellular Ca^2+^ levels and cAMP signaling. None of the three drugs showed an effect alone, but in double and triple therapy they showed synergistic effects, with metoprolol being the most effective for photoreceptors^65^. As we analyzed taurine, betaxolol and brimonidine, which are all involved in GPCR signaling pathways, e.g. betaxolol inhibits NADPH oxidase to reduce superoxide radicals via Gs-coupled GPCRs, and brimonidine and taurine inhibit cAMP production and Ca^2+^ channels via Gi-coupled GCPRs, the protective effect described by Leinonen and colleagues is comparable to our experiments^65^. Betaxolol and taurine were effective, whereas brimonidine alone showed the least protective effect. This could be due to the individual effect of the drugs, and future study will focus on the synergistic effects.

Another example of a beta-adrenergic receptor blocker is metipranolol, which inhibited nitrosative stress resulting in protection of photoreceptors in *rd10* mice^66^. In another study, betaxolol, timolol and nipradilol were reported to be protective against hypoxia-induced cell death in a viability assay using rat RGCs^67^. Some Ca^2+^ channel blockers, such as nilvadipine and nicardipine, preserved photoreceptor cells in *rd* mice and may be used to treat RP patients^68^. Physiological in vivo studies showed that luteolin and pentazocine have neuroprotective effects on photoreceptors in *rd10* mice; however, the effect on RGCs was not investigated^69,70^.

As blindness cannot be adequately treated or cured, it contributes to a deterioration in the quality of life of patients and represents a burden to society^62^. The drugs tested in this study are promising candidates for the treatment of degenerative retinal diseases and should be further investigated, particularly in combination with other RP treatments, such as retinal prostheses. The aim would be to use a combined therapy to improve the results of previous retinal implants, which have fallen short of expectations. Possible dosage forms include locally applied eye drops or nanosuspensions for brimonidine^71^ and betaxolol^72^ or systemic intake via drinking water for taurine^29^. Another delivery option involves integrating fluid reservoirs into retinal implants^73^. Prior pharmacological studies must define the therapeutically effective concentrations of the individual substances. These experiments are an important first step towards improving the remaining RGC function in degenerated retinas, halting the degenerative process or suppressing the subsequent remodeling process and towards a clinical approach, as many patients go blind due to retinal degeneration and need optimized treatment options. Furthermore, the search for a therapeutic option for patients who are not yet totally blind and suffer from partial visual loss may be better addressed with drugs than with gene therapy, prosthetics, or stem cells as they do not yet meet the inclusion criteria for advanced vision loss for these therapies^6,7,65,74^. As suggested by Leinonen and colleagues as well as in the review of Maneu, multi-target therapy may be more protective in complex diseases^62,65^. Therefore, ex vivo experiments with a combination of taurine, brimonidine, and betaxolol are planned to analyze a possible synergistic effect, as well as in vivo experiments to investigate the effects of long-time application.

## Methods

### Animals

Male and female B6.CXB1-Pde6brd10/J (*rd10*) mice were bred at the Institute of Laboratory Animal Science (Faculty of Medicine, RWTH Aachen University, Aachen, Germany). The source of the parental mice was the Jackson Laboratory (Bar Harbor, ME, USA). All animals were kept under controlled light conditions (12:12 h light/dark cycle) at RT of 21–23 °C and humidity of 35–65%. Water and food were provided ad libitum, and cages were cleaned once a week. At 3- to 4-months of age, mice were deeply anesthetized and sacrificed with an overdose of isoflurane (AbbVie, Wiesbaden, Germany). All experiments were performed in accordance with the ARVO Statement for the Use of Animals in Ophthalmic and Vision Research and the German Animal Welfare Act. The study was approved by the regulatory authorities (Institute of Laboratory Animal Science, Faculty of Medicine, RWTH Aachen University, reference number: 50095A4) and in accordance with the ARRIVE guidelines^75^.

### Medium

Ames’ medium^76^ (Sigma-Aldrich, St. Louis, MO) was prepared as previously described^55^. After dissolving in water, gassing with 100% CO_2_ for 30 min at RT, and supplementation with sodium bicarbonate, the solution was adjusted to a pH of 7.4–7.5 with sodium hydroxide and gassed continuously with carbogen gas (95% O_2_, 5% CO_2_). Taurine, brimonidine and betaxolol were dissolved in Ames’ medium and used at concentrations of 1.0 and 1.5 mM^41,55,77,78^, 150 µM^32,79,80^ and 100 µM^79,80^, respectively, based on corresponding literature data.

### Retina preparation

Retinas were prepared as previously described^55,56^. After sacrifice, both eyes of an animal were enucleated and placed in freshly carbogenized Ames’ medium. The left eye was punctured at the limbus, cut halfway along the radial axis, and stored in freshly carbogenized Ames’ medium for later use. The right eye was opened by a circumferential incision and the anterior segment and lens were removed. The retina was carefully detached from the eyecup, separated by a cut through the optic nerve, and completely freed from the vitreous body using forceps. The retina was cut into a square, placed on a nitrocellulose frame with the RGCs facing up, and then placed upside down on the electrode field of a MEA.

### Multielectrode Array (MEA) Setup

The MEA2100 system from Multi Channel Systems (Reutlingen, Germany) was used for electrophysiological recordings on the mouse retina. It consists of a headstage with an integrated preamplifier for recording and stimulation, and an interface board that serves as a digital-to-analog converter for real-time data transfer. The MEA was inserted into the headstage, which was connected to the interface board, which was connected to a personal computer (PC). The MEA setup was placed on an air-suspended table in a Faraday cage (Ametek, Berwyn, PA) to minimize noise from vibrations and surrounding electronic equipment.

MEAs of the type 60MEA200/30iR-Ti-pr-T (Multi Channel Systems) with 60 titanium nitride (TiN) electrodes surrounded by a plastic ring were used. The electrodes, 30 µm in diameter and 200 µm apart, were arranged in a grid of 8 × 8 electrodes, with four electrodes missing at the corners. One electrode served as an internal reference, and the other 59 electrodes were used for recording and stimulation. Before each experiment, the MEAs were hydrophilized with oxygen plasma at 0.5 mbar for 2 min in a plasma cleaner (Diener Electronic, Ebhausen, Germany) to improve contact between the tissue and the electrodes. Throughout the experiment, the retina was perfused with fresh, continuously carbogenized Ames’ medium at a flow rate of 2.5 mL/min at RT using a gravity-based perfusion system (VC^3^, ALA Scientific Instruments, Farmingdale, NY).

### Experimental protocol

Perfusion experiments with taurine, betaxolol, and brimonidine were performed according to a standardized protocol. Each experiment started with a 40-min lead time to establish constant experimental conditions, i.e., to allow the retina to recover from the preparation stress and to achieve stabilized electrical activity. Fresh, continuously carbogenized Ames’ medium plus taurine, betaxolol, or brimonidine (at the appropriate concentrations) was then applied to the retina for 40 min via the perfusion system. This application was repeated after an intermediate washing step for 40 min with Ames’ medium. A final wash with Ames’ medium was performed for another 40 min (Supplementary Fig. S4).

During the different perfusion steps, the electrical activity of the RGCs was recorded at specific times using the Multi Channel Experimenter software (Multi Channel Systems) at a sampling rate of 25 kHz (Supplementary Fig. S4). The raw data were filtered with a second-order Butterworth high-pass (cut-off at 200 Hz) and low-pass filter (cut-off at 2 kHz; (Supplementary Fig. S4)). For analyzing the LFPs, a 50 Hz low pass filter was applied. A spike detection threshold of -20 µV was then set to detect action potentials (APs), except for noisy channels where the threshold was manually adjusted. Measurements of 70 s each were performed to record the spontaneous firing frequency of the cells or their response to an electrical stimulation pulse.

For each recording, one or two electrodes were selected as stimulation electrodes, but stimulation was always applied to only one electrode at a time. A biphasic current pulse (± 40–100 µA, 500 µs per phase) was used, with the cathodic phase preceding the anodic phase^81,82^. Seven pulses were delivered per 70-s measurement with an interstimulus interval of 10 s, starting with the first pulse at 5 s. The eight adjacent electrodes were selected as recording electrodes for further analysis.

### Data analysis

Detected APs were assigned to individual RGCs using Offline Sorter software 4 (Plexon Inc, Dallas, TX) by sorting and clustering spikes according to their distinct waveforms using principal component analysis (PCA). Clearly separated waveforms were combined and labeled as units representing individual cells. Spikes that could not be clearly identified were not classified, and spikes at the origin of the PCA coordinate system were not sorted because they do not clearly represent a single cell (Supplementary Fig. S4). The following settings were selected by default: at least 40 waveforms were required to define a unit; for spontaneous recordings, all channels were sorted except for high background noise; for stimulation recordings, only the eight channels adjacent to the stimulation electrode were sorted.

Data were further processed using NeuroExplorer software 5 (Nex Technologies, Colorado Springs, CO) to assess spontaneous firing frequency, excitability following electrical stimulation, bursting behavior, and oscillations (Supplementary Fig. S4). Data were exported to Excel and a custom written Python script^56^ was used to partially automate data analysis. The spike response rate to an electrical stimulus was calculated as the coefficient of the firing rate 3 s before the stimulus and the firing rate 0.5 s after the stimulus and was defined as the stimulation efficiency (spike response coefficient). A burst analysis identified spikes in bursts with the following interval algorithm parameters: maximum interval of 0.01 s for the start of a burst and 0.03 s for the end of a burst, minimum interval of 0.02 s between bursts, minimum duration of a burst of 0.01 s, and a minimum number of three spikes within a burst^56^. A power spectral density calculation including a fast Fourier transform was used to calculate the main oscillation frequency in the LFPs for each channel. Channels showing no spontaneous activity and no oscillations during all perfusion steps were excluded from the analysis. Analysis of spontaneous activity was performed on cell level and on channel level according to previous studies^55,56^. Spike sorting precedes cell level analysis to analyze the behavior of a single cell over time. Due to spike sorting, many spikes remained unsorted and, thus, a lot of information would not have been considered. Therefore, analysis on channel level was performed as well without spike sorting procedure to include almost all measured data. The frequency at channel level represents spikes of more than one cell.

### Experimental design

For each drug, 4–5 *rd10* retinas were used (for 1.0 mM taurine, n = 4 retinas; for 1.5 mM taurine, brimonidine and betaxolol, n = 5 retinas each). To minimize the total number of animals, separate control retinas were not used; instead, the first perfusion step with Ames’ medium (A.1^st^), lasting 40 min in each experiment, served as control. All groups included both male and female animals. Five retinas were discarded because they did not meet the experimental criteria. A total of 19 *rd10* mice (8 male, 11 female) were used for MEA recordings.

### Statistical analysis

GraphPad Prism software 10.4.1 (GraphPad Software, Boston, MA) was used for graph generation and statistical analysis. A one-way ANOVA with Tukey’s multiple comparisons test was performed on all data sets presented, except for the comparison of the two taurine concentrations, for which a two-tailed t-test was used. A *p* value < 0.05 was considered significant. In the figures, the different levels of significance are indicated by asterisks: *p < 0.05, **p < 0.01, ***p < 0.001, and ****p < 0.0001. Unless otherwise stated, data are presented as mean ± standard deviation. When presenting the data as box-and-whisker plot, Tukey method was used to display the whiskers and outliers, with the mean values are marked with a + .

## Electronic supplementary material

Below is the link to the electronic supplementary material.

Supplementary Material 1

## Data Availability

The datasets generated and/or analysed during the current study are not publicly available due to further analyses within the institution but are available from the corresponding author on reasonable request.

## References

[1] Boughman, J. A., Conneally, P. M. & Nance, W. E. Population genetic studies of retinitis pigmentosa. *Am. J. Hum. Genet.* **32**, 223–235 (1980). 7386458 PMC1686021

[2] Pagon, R. A. Retinitis pigmentosa. *Surv. Ophthalmol.* **33**, 137–177. 10.1016/0039-6257(88)90085-9 (1988). 3068820 10.1016/0039-6257(88)90085-9

[3] Miyata, M. et al. Long-term efficacy and safety of anti-VEGF therapy in retinitis pigmentosa: A case report. *BMC Ophthalmol.* **18**, 248. 10.1186/s12886-018-0914-z (2018). 30217183 10.1186/s12886-018-0914-z PMC6137720

[4] Sivakumar, P. et al. Barriers in utilisation of low vision assistive products. *Eye (Lond)*. **34**, 344–351. 10.1038/s41433-019-0545-5 (2020). 31388131 10.1038/s41433-019-0545-5 PMC7002618

[5] Nguyen, X. T. et al. Outcome of cataract surgery in patients with retinitis pigmentosa. *Am. J. Ophthalmol.* **246**, 1–9. 10.1016/j.ajo.2022.10.001 (2023). 36252678 10.1016/j.ajo.2022.10.001

[6] Ayton, L. N. et al. An update on retinal prostheses. *Clin. Neurophysiol.* **131**, 1383–1398. 10.1016/j.clinph.2019.11.029 (2020). 31866339 10.1016/j.clinph.2019.11.029 PMC7198351

[7] Stingl, K. et al. Therapy with voretigene neparvovec. How to measure success? *Prog Retin Eye Res.* **92**, 101115. 10.1016/j.preteyeres.2022.101115 (2023). 36096933 10.1016/j.preteyeres.2022.101115

[8] Wong, W. L. et al. Global prevalence of age-related macular degeneration and disease burden projection for 2020 and 2040: A systematic review and meta-analysis. *Lancet Glob Health*. **2**, e106–116. 10.1016/S2214-109X(13)70145-1 (2014). 25104651 10.1016/S2214-109X(13)70145-1

[9] Dadgostar, H. & Waheed, N. The evolving role of vascular endothelial growth factor inhibitors in the treatment of neovascular age-related macular degeneration. *Eye (Lond)*. **22**, 761–767. 10.1038/eye.2008.86 (2008). 18388961 10.1038/eye.2008.86

[10] Schachar, I. H. Concerning syfovre approval for geographic atrophy. *JAMA Ophthalmol.* **142**, 85–86. 10.1001/jamaophthalmol.2023.5584 (2024). 38060249 10.1001/jamaophthalmol.2023.5584

[11] Kang, C. Avacincaptad pegol: First approval. *Drugs* **83**, 1447–1453. 10.1007/s40265-023-01948-8 (2023). 37814173 10.1007/s40265-023-01948-8

[12] Liakopoulos, S., von der Emde, L., Biller, M. L., Ach, T. & Holz, F. G. Geographic atrophy in age-related macular degeneration. *Dtsch. Arztebl Int.*10.3238/arztebl.m2025.0003 (2025). 39836449 10.3238/arztebl.m2025.0003 PMC12434720

[13] Muqit, M. M. K. et al. Prosthetic visual acuity with the PRIMA subretinal microchip in patients with atrophic age-related macular degeneration at 4 years Follow-up. *Ophthalmol. Sci.* **4**, 100510. 10.1016/j.xops.2024.100510 (2024). 38881600 10.1016/j.xops.2024.100510 PMC11179408

[14] Marc, R. E., Jones, B. W., Watt, C. B. & Strettoi, E. Neural remodeling in retinal degeneration. *Prog Retin Eye Res.* **22**, 607–655 (2003). 12892644 10.1016/s1350-9462(03)00039-9

[15] Jones, B. W. et al. Retinal remodeling in human retinitis pigmentosa. *Exp. Eye Res.* **150**, 149–165. 10.1016/j.exer.2016.03.018 (2016). 27020758 10.1016/j.exer.2016.03.018 PMC5031517

[16] Jones, B. W. et al. Retinal remodeling and metabolic alterations in human AMD. *Front. Cell. Neurosci.* **10**, 103. 10.3389/fncel.2016.00103 (2016). 27199657 10.3389/fncel.2016.00103 PMC4848316

[17] Ma, D. J. in *Inherited Retinal Disease* (ed Hyeong-Gon Yu) Ch. Chapter 1, 1–19 (Springer, 2022).

[18] Chang, B. et al. Two mouse retinal degenerations caused by missense mutations in the beta-subunit of rod cGMP phosphodiesterase gene. *Vis. Res.* **47**, 624–633. 10.1016/j.visres.2006.11.020 (2007). 17267005 10.1016/j.visres.2006.11.020 PMC2562796

[19] Biswas, S. et al. Pharmacological analysis of intrinsic neuronal oscillations in rd10 retina. *PLoS One*. **9**, e99075. 10.1371/journal.pone.0099075 (2014). 24918437 10.1371/journal.pone.0099075 PMC4053359

[20] Haselier, C. et al. Correlations between specific patterns of spontaneous activity and stimulation efficiency in degenerated retina. *PLoS One*. **12**, e0190048. 10.1371/journal.pone.0190048 (2017). 29281713 10.1371/journal.pone.0190048 PMC5744965

[21] Gehlen, J. et al. Blockade of retinal oscillations by benzodiazepines improves efficiency of electrical stimulation in the mouse model of RP, rd10. *Invest. Ophthalmol. Vis. Sci.* **61**, 37. 10.1167/iovs.61.13.37 (2020). 33252632 10.1167/iovs.61.13.37 PMC7705397

[22] Haq, W., Arango-Gonzalez, B., Zrenner, E., Euler, T. & Schubert, T. Synaptic remodeling generates synchronous oscillations in the degenerated outer mouse retina. *Front. Neural Circuits*. **8**, 108. 10.3389/fncir.2014.00108 (2014). 25249942 10.3389/fncir.2014.00108 PMC4155782

[23] Borowska, J., Trenholm, S. & Awatramani, G. B. An intrinsic neural oscillator in the degenerating mouse retina. *J. Neurosci.* **31**, 5000–5012. 10.1523/JNEUROSCI.5800-10.2011 (2011). 21451038 10.1523/JNEUROSCI.5800-10.2011 PMC6622979

[24] Trenholm, S. et al. Intrinsic oscillatory activity arising within the electrically coupled AII amacrine-ON cone bipolar cell network is driven by voltage-gated Na + channels. *J. Physiol.* **590**, 2501–2517. 10.1113/jphysiol.2011.225060 (2012). 22393249 10.1113/jphysiol.2011.225060 PMC3424767

[25] Choi, H. et al. Intrinsic bursting of AII Amacrine cells underlies oscillations in the rd1 mouse retina. *J. Neurophysiol.* **112**, 1491–1504. 10.1152/jn.00437.2014 (2014). 25008417 10.1152/jn.00437.2014 PMC4137253

[26] Bagli, E., Goussia, A., Moschos, M. M., Agnantis, N. & Kitsos, G. Natural compounds and neuroprotection: Mechanisms of action and novel delivery systems. *Vivo* **30**, 535–547 (2016). 27566070

[27] Schaffer, S. & Kim, H. W. Effects and mechanisms of taurine as a therapeutic agent. *Biomol. Ther. (Seoul)*. **26**, 225–241. 10.4062/biomolther.2017.251 (2018). 29631391 10.4062/biomolther.2017.251 PMC5933890

[28] Tao, Y. et al. Systemic taurine treatment provides neuroprotection against retinal photoreceptor degeneration and visual function impairments. *Drug Des. Devel Ther.* **13**, 2689–2702. 10.2147/DDDT.S194169 (2019). 31496648 10.2147/DDDT.S194169 PMC6689665

[29] Martinez-Vacas, A. et al. Systemic taurine treatment affords functional and morphological neuroprotection of photoreceptors and restores retinal pigment epithelium function in RCS rats. *Redox Biol.* **57**, 102506. 10.1016/j.redox.2022.102506 (2022). 36270186 10.1016/j.redox.2022.102506 PMC9583577

[30] Garcia-Ayuso, D. et al. Taurine: A promising nutraceutic in the prevention of retinal degeneration. *Neural Regen Res.* **19**, 606–610. 10.4103/1673-5374.380820 (2024). 37721291 10.4103/1673-5374.380820 PMC10581579

[31] Fan, S., Agrawal, A., Gulati, V., Neely, D. G. & Toris, C. B. Daytime and nighttime effects of brimonidine on IOP and aqueous humor dynamics in participants with ocular hypertension. *J. Glaucoma*. **23**, 276–281. 10.1097/IJG.0000000000000051 (2014). 24886701 10.1097/IJG.0000000000000051 PMC4435779

[32] Nizari, S. et al. Non-amyloidogenic effects of alpha2 adrenergic agonists: Implications for brimonidine-mediated neuroprotection. *Cell. Death Dis.* **7**, e2514. 10.1038/cddis.2016.397 (2016). 27929541 10.1038/cddis.2016.397 PMC5260990

[33] Conti, F. et al. Brimonidine is neuroprotective in animal paradigm of retinal ganglion cell damage. *Front. Pharmacol.* **12**, 705405. 10.3389/fphar.2021.705405 (2021). 34366858 10.3389/fphar.2021.705405 PMC8333612

[34] Valiente-Soriano, F. J. et al. Topical brimonidine or intravitreal BDNF, CNTF, or bFGF protect cones against phototoxicity. *Transl Vis. Sci. Technol.* **8**, 36. 10.1167/tvst.8.6.36 (2019). 31890348 10.1167/tvst.8.6.36 PMC6919195

[35] Rajagopalan, L. et al. A nonhuman primate model of blue light-induced progressive outer retina degeneration showing brimonidine drug delivery system-mediated cyto- and neuroprotection. *Exp. Eye Res.* **209**, 108678. 10.1016/j.exer.2021.108678 (2021). 34153289 10.1016/j.exer.2021.108678

[36] Freeman, W. R. et al. Randomized phase IIb study of brimonidine drug delivery system generation 2 for geographic atrophy in Age-Related macular degeneration. *Ophthalmol. Retina*. **7**, 573–585. 10.1016/j.oret.2023.03.001 (2023). 36906177 10.1016/j.oret.2023.03.001

[37] Stamper, R. L., Lieberman, M. F. & Drake, M. V. in *Becker-Shaffer’s Diagnosis and Therapy of the Glaucomas* (eds R. L. Stamper, M. F. Lieberman, & M. V. Drake) 392–406 (Mosby, 2009).

[38] Osborne, N. N., Cazevieille, C., Carvalho, A. L., Larsen, A. K. & DeSantis, L. In vivo and in vitro experiments show that betaxolol is a retinal neuroprotective agent. *Brain Res.* **751**, 113–123. 10.1016/s0006-8993(96)01393-5 (1997). 9098574 10.1016/s0006-8993(96)01393-5

[39] Agarwal, N. et al. Levobetaxolol-induced up-regulation of retinal bFGF and CNTF mRNAs and preservation of retinal function against a photic-induced retinopathy. *Exp. Eye Res.* **74**, 445–453. 10.1006/exer.2001.1145 (2002). 12076088 10.1006/exer.2001.1145

[40] Gargini, C., Terzibasi, E., Mazzoni, F. & Strettoi, E. Retinal organization in the retinal degeneration 10 (rd10) mutant mouse: A morphological and ERG study. *J. Comp. Neurol.* **500**, 222–238. 10.1002/cne.21144 (2007). 17111372 10.1002/cne.21144 PMC2590657

[41] Froger, N. et al. Taurine provides neuroprotection against retinal ganglion cell degeneration. *PLoS One*. **7**, e42017. 10.1371/journal.pone.0042017 (2012). 23115615 10.1371/journal.pone.0042017 PMC3480351

[42] Metoki, T. et al. Study of effects of antiglaucoma eye drops on N-methyl-D-aspartate-induced retinal damage. *Jpn J. Ophthalmol.* **49**, 453–461. 10.1007/s10384-005-0253-5 (2005). 16365790 10.1007/s10384-005-0253-5

[43] Goo, Y. S., Park, D. J., Ahn, J. R. & Senok, S. S. Spontaneous oscillatory rhythms in the degenerating mouse retina modulate retinal ganglion cell responses to electrical stimulation. *Front. Cell. Neurosci.* **9**, 512. 10.3389/fncel.2015.00512 (2015). 26793063 10.3389/fncel.2015.00512 PMC4709854

[44] Yee, C. W., Toychiev, A. H. & Sagdullaev, B. T. Network deficiency exacerbates impairment in a mouse model of retinal degeneration. *Front. Syst. Neurosci.* **6**, 8. 10.3389/fnsys.2012.00008 (2012). 22383900 10.3389/fnsys.2012.00008 PMC3285818

[45] Toychiev, A. H., Ivanova, E., Yee, C. W. & Sagdullaev, B. T. Block of gap junctions eliminates aberrant activity and restores light responses during retinal degeneration. *J. Neurosci.* **33**, 13972–13977. 10.1523/JNEUROSCI.2399-13.2013 (2013). 23986234 10.1523/JNEUROSCI.2399-13.2013 PMC3756747

[46] Ivanova, E., Yee, C. W., Baldoni, R. Jr. & Sagdullaev, B. T. Aberrant activity in retinal degeneration impairs central visual processing and relies on Cx36-containing gap junctions. *Exp. Eye Res.* **150**, 81–89. 10.1016/j.exer.2015.05.013 (2016). 26005040 10.1016/j.exer.2015.05.013 PMC4655183

[47] Xiang, Z. et al. Inhibition of non-NMDA ionotropic glutamate receptors delays the retinal degeneration in rd10 mouse. *Neuropharmacology* **139**, 137–149. 10.1016/j.neuropharm.2018.06.027 (2018). 29940208 10.1016/j.neuropharm.2018.06.027

[48] Barrett, J. M., Degenaar, P. & Sernagor, E. Blockade of pathological retinal ganglion cell hyperactivity improves optogenetically evoked light responses in rd1 mice. *Front. Cell. Neurosci.* **9**, 330. 10.3389/fncel.2015.00330 (2015). 26379501 10.3389/fncel.2015.00330 PMC4548307

[49] Jia, F. et al. Taurine is a potent activator of extrasynaptic GABA(A) receptors in the thalamus. *J. Neurosci.* **28**, 106–115. 10.1523/JNEUROSCI.3996-07.2008 (2008). 18171928 10.1523/JNEUROSCI.3996-07.2008 PMC6671153

[50] Hadj-Said, W. et al. Taurine promotes retinal ganglion cell survival through GABAB receptor activation. *Adv. Exp. Med. Biol.* **975 Pt 2**, 687–701. 10.1007/978-94-024-1079-2_54 (2017). 28849492 10.1007/978-94-024-1079-2_54

[51] Zhou, X., Zhang, T. & Wu, J. Brimonidine enhances inhibitory postsynaptic activity of OFF- and ON-type retinal ganglion cells in a Wistar rat chronic glaucoma model. *Exp. Eye Res.* **189**, 107833. 10.1016/j.exer.2019.107833 (2019). 31618613 10.1016/j.exer.2019.107833

[52] Gross, R. L., Hensley, S. H., Gao, F. & Wu, S. M. Retinal ganglion cell dysfunction induced by hypoxia and glutamate: Potential neuroprotective effects of beta-blockers. *Surv. Ophthalmol.* **43** (Suppl 1), S162–170 (1999). 10416759 10.1016/s0039-6257(99)00054-5

[53] Moore, B. D., Kiley, C. W., Sun, C. & Usrey, W. M. Rapid plasticity of visual responses in the adult lateral geniculate nucleus. *Neuron* **71**, 812–819. 10.1016/j.neuron.2011.06.025 (2011). 21903075 10.1016/j.neuron.2011.06.025 PMC3170518

[54] Alitto, H., Rathbun, D. L., Vandeleest, J. J., Alexander, P. C. & Usrey, W. M. The augmentation of retinogeniculate communication during thalamic burst mode. *J. Neurosci.* **39**, 5697–5710. 10.1523/JNEUROSCI.2320-18.2019 (2019). 31109958 10.1523/JNEUROSCI.2320-18.2019 PMC6636080

[55] Ingensiep, C., Schaffrath, K., Denecke, B., Walter, P. & Johnen, S. A multielectrode array-based hypoxia model for the analysis of electrical activity in murine retinae. *J. Neurosci. Res.* **99**, 2172–2187. 10.1002/jnr.24899 (2021). 34110645 10.1002/jnr.24899

[56] Ingensiep, C., Schaffrath, K., Walter, P. & Johnen, S. Effects of hydrostatic pressure on electrical retinal activity in a multielectrode array-based ex vivo Glaucoma acute model. *Front. Neurosci.* **16**, 831392. 10.3389/fnins.2022.831392 (2022). 35177963 10.3389/fnins.2022.831392 PMC8845467

[57] Ahn, J. et al. Correlated activity in the degenerate retina inhibits focal response to electrical stimulation. *Front. Cell. Neurosci.* **16**, 889663. 10.3389/fncel.2022.889663 (2022). 35602554 10.3389/fncel.2022.889663 PMC9114441

[58] Rimmele, T. et al. What blood temperature for an ex vivo extracorporeal circuit? *Artif. Organs*. **35**, 593–601. 10.1111/j.1525-1594.2010.01147.x (2011). 21314837 10.1111/j.1525-1594.2010.01147.x PMC3224854

[59] Opitz, A., Falchier, A., Linn, G. S., Milham, M. P. & Schroeder, C. E. Limitations of ex vivo measurements for in vivo neuroscience. *Proc. Natl. Acad. Sci. U S A*. **114**, 5243–5246. 10.1073/pnas.1617024114 (2017). 28461475 10.1073/pnas.1617024114 PMC5441777

[60] Barhoum, R. et al. Functional and structural modifications during retinal degeneration in the rd10 mouse. *Neuroscience* **155**, 698–713. 10.1016/j.neuroscience.2008.06.042 (2008). 18639614 10.1016/j.neuroscience.2008.06.042

[61] Cha, S. et al. Stage-dependent changes of visual function and electrical response of the retina in the rd10 mouse model. *Front. Cell. Neurosci.* **16**, 926096. 10.3389/fncel.2022.926096 (2022). 35936494 10.3389/fncel.2022.926096 PMC9345760

[62] Maneu, V., Lax, P., De Diego, A. M. G., Cuenca, N. & Garcia, A. G. Combined drug triads for synergic neuroprotection in retinal degeneration. *Biomed. Pharmacother*. **149**, 112911. 10.1016/j.biopha.2022.112911 (2022). 36068774 10.1016/j.biopha.2022.112911

[63] Chen, Y. et al. Synergistically acting agonists and antagonists of G protein-coupled receptors prevent photoreceptor cell degeneration. *Sci. Signal.* **9**, ra74. 10.1126/scisignal.aag0245 (2016). 27460988 10.1126/scisignal.aag0245 PMC4972460

[64] Chen, Y. & Palczewski, K. Systems pharmacology links GPCRs with retinal degenerative disorders. *Annu. Rev. Pharmacol. Toxicol.* **56**, 273–298. 10.1146/annurev-pharmtox-010715-103033 (2016). 25839098 10.1146/annurev-pharmtox-010715-103033 PMC4580525

[65] Leinonen, H. et al. A combination treatment based on drug repurposing demonstrates mutation-agnostic efficacy in pre-clinical retinopathy models. *Nat. Commun.* **15**, 5943. 10.1038/s41467-024-50033-5 (2024). 39009597 10.1038/s41467-024-50033-5 PMC11251169

[66] Kanan, Y. et al. Metipranolol promotes structure and function of retinal photoreceptors in the rd10 mouse model of human retinitis pigmentosa. *J. Neurochem*. **148**, 307–318. 10.1111/jnc.14613 (2019). 30315650 10.1111/jnc.14613 PMC12554146

[67] Chen, Y. N. et al. Hypoxia-induced retinal ganglion cell death and the neuroprotective effects of beta-adrenergic antagonists. *Brain Res.* **1148**, 28–37. 10.1016/j.brainres.2007.02.027 (2007). 17368577 10.1016/j.brainres.2007.02.027

[68] Takano, Y. et al. Study of drug effects of calcium channel blockers on retinal degeneration of Rd mouse. *Biochem. Biophys. Res. Commun.* **313**, 1015–1022. 10.1016/j.bbrc.2003.12.034 (2004). 14706644 10.1016/j.bbrc.2003.12.034

[69] Liu, X. B. et al. Luteolin delays photoreceptor degeneration in a mouse model of retinitis pigmentosa. *Neural Regen Res.* **16**, 2109–2120. 10.4103/1673-5374.303537 (2021). 33642401 10.4103/1673-5374.303537 PMC8343326

[70] Wang, J., Xiao, H., Barwick, S., Liu, Y. & Smith, S. B. Optimal timing for activation of Sigma 1 receptor in the Pde6b(rd10)/J (rd10) mouse model of retinitis pigmentosa. *Exp. Eye Res.* **202**, 108397. 10.1016/j.exer.2020.108397 (2021). 33310057 10.1016/j.exer.2020.108397 PMC7808329

[71] Khopade, A. J. et al. Preclinical evaluation of a novel once-a-day brimonidine ophthalmic nanosuspension. *J. Ocul Pharmacol. Ther.*10.1089/jop.2023.0038 (2023). 37646731 10.1089/jop.2023.0038

[72] Hu, J. et al. Critical evaluation of multifunctional betaxolol hydrochloride nanoformulations for effective sustained intraocular pressure reduction. *Int. J. Nanomed.* **17**, 5915–5931. 10.2147/IJN.S382968 (2022). 10.2147/IJN.S382968 PMC9729687 36506343

[73] Wu, J. et al. Progress on designing a chemical retinal prosthesis. *Front. Cell. Neurosci.* **16**, 898865. 10.3389/fncel.2022.898865 (2022). 35774083 10.3389/fncel.2022.898865 PMC9239740

[74] da Cruz, L. et al. Phase 1 clinical study of an embryonic stem cell-derived retinal pigment epithelium patch in age-related macular degeneration. *Nat. Biotechnol.* **36**, 328–337. 10.1038/nbt.4114 (2018). 29553577 10.1038/nbt.4114

[75] du Percie, N. et al. Reporting animal research: Explanation and elaboration for the ARRIVE guidelines 2.0. *PLoS Biol.* **18**, e3000411. 10.1371/journal.pbio.3000411 (2020). 32663221 10.1371/journal.pbio.3000411 PMC7360025

[76] Ames, A. & Nesbett, F. B. In vitro retina as an experimental model of the central nervous system. *J. Neurochem*. **37**, 867–877. 10.1111/j.1471-4159.1981.tb04473.x (1981). 7320727 10.1111/j.1471-4159.1981.tb04473.x

[77] Hernández-Benítez, R., Ramos-Mandujano, G. & Pasantes-Morales, H. Taurine stimulates proliferation and promotes neurogenesis of mouse adult cultured neural stem/progenitor cells. *Stem Cell. Res.* **9**, 24–34. 10.1016/j.scr.2012.02.004 (2012). 22484511 10.1016/j.scr.2012.02.004

[78] Chen, K. et al. Taurine protects transformed rat retinal ganglion cells from hypoxia-induced apoptosis by preventing mitochondrial dysfunction. *Brain Res.* **1279**, 131–138. 10.1016/j.brainres.2009.04.054 (2009). 19427840 10.1016/j.brainres.2009.04.054

[79] Bull, N. D. et al. Use of an adult rat retinal explant model for screening of potential retinal ganglion cell neuroprotective therapies. *Invest. Ophthalmol. Vis. Sci.* **52**, 3309–3320. 10.1167/iovs.10-6873 (2011). 21345987 10.1167/iovs.10-6873 PMC3109030

[80] Melena, J., Stanton, D. & Osborne, N. N. Comparative effects of antiglaucoma drugs on voltage-dependent calcium channels. *Graefes Arch. Clin. Exp. Ophthalmol.* **239**, 522–530. 10.1007/s004170100312 (2001). 11521697 10.1007/s004170100312

[81] McIntyre, C. C. & Grill, W. M. Selective microstimulation of central nervous system neurons. *Ann. Biomed. Eng.* **28**, 219–233. 10.1114/1.262 (2000). 10784087 10.1114/1.262

[82] Merrill, D. R., Bikson, M. & Jefferys, J. G. Electrical stimulation of excitable tissue: Design of efficacious and safe protocols. *J. Neurosci. Methods*. **141**, 171–198. 10.1016/j.jneumeth.2004.10.020 (2005). 15661300 10.1016/j.jneumeth.2004.10.020

