# Supplementary material for: Effects of taurine, brimonidine and betaxolol on oscillation modulation and stimulation efficiency in degenerated rd10 mouse retinas
Source: Sci Rep. 2025 Jun 20;15:20209. doi: 10.1038/s41598-025-06440-9 (PMC12181435; doi:10.1038/s41598-025-06440-9)
Supplement: Supplementary file 1 — Supplementary Material 1 [file 41598_2025_6440_MOESM1_ESM.pdf]

Supplementary information

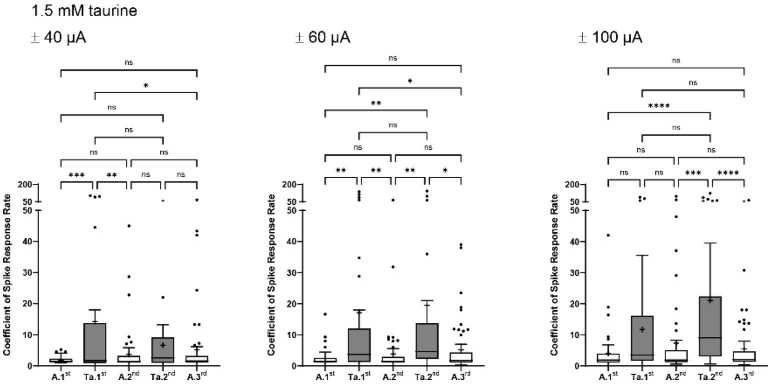

Figure S1. Effect of taurine on the stimulation efficiency of *rd10* neurons to electrical stimulation. Analysis of the stimulation efficiency (coefficient of spike response rate) of *rd10* neurons to electrical stimulation (± 40 μA, ± 60 μA and ± 100 μA, 500 μs per phase) during the individual perfusion steps with Ames' medium (A.1st – A.3rd, white) and 1.5 mM taurine (Ta.1st – Ta.2nd, dark grey). Data are presented as box-and-whisker plots (27-61 cells analyzed for ± 40 μA; 22-60 cells analyzed for ± 60 μA; 34-73 cells analyzed for ± 100 μA).

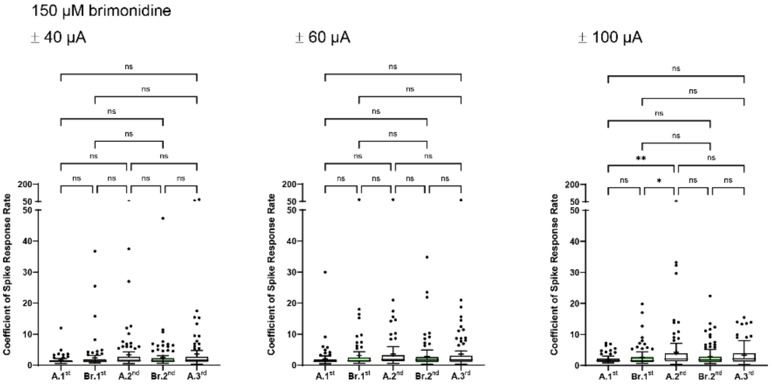

Figure S2. Effect of brimonidine on the stimulation efficiency of *rd10* neurons to electrical stimulation. Analysis of the stimulation efficiency of *rd10* neurons to electrical stimulation (± 40 μA, ± 60 μA and ± 100 μA, 500 μs per phase) during the individual perfusion steps with Ames' medium (A.1st – A.3rd, white) and 150 μM brimonidine (Br.1st – Br.2nd, light green). Data are presented as box-and-whisker plots (79-115 cells analyzed for ± 40 μA; 82-114 cells analyzed for ± 60 μA; 79-114 cells analyzed for ± 100 μA).

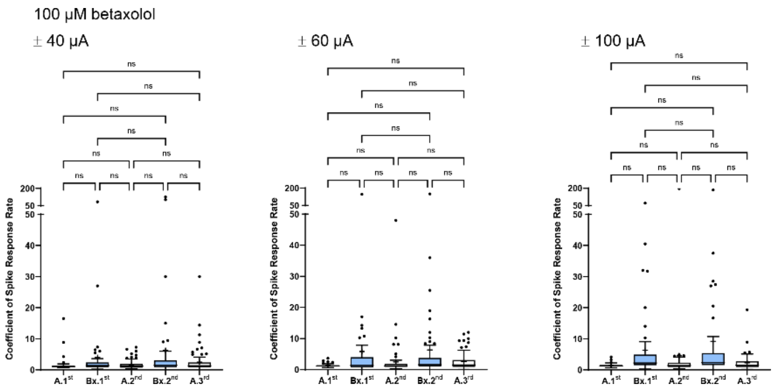

Figure S3. Effect of betaxolol on the stimulation efficiency of *rd10* neurons to electrical stimulation. Analysis of the stimulation efficiency of *rd10* neurons to electrical stimulation (± 40 µA, ± 60 µA and ± 100 µA, 500 µs per phase) during the individual perfusion steps with Ames' medium (A.1st – A.3rd, white) and 100 µM betaxolol (Bx.1st – Bx.2nd, light blue). Data are presented as box-and-whisker plots (41-81 cells analyzed for ± 40 µA; 38-76 cells analyzed for ± 60 µA; 43-72 cells analyzed for ± 100 µA).

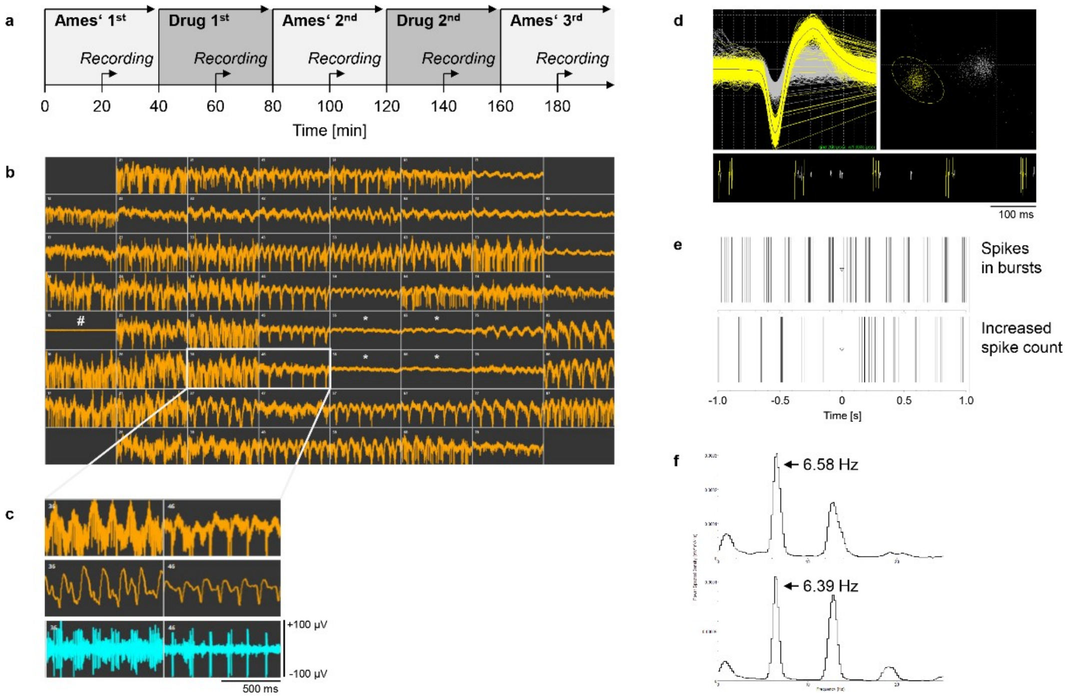

Figure S4. Time course of MEA experiments and examples of recorded and analyzed electrical activity from *rd10* retinas. (a) Experiments were standardized and started with 40 min perfusion with Ames' medium (Ames' 1st) to establish constant conditions and record baseline retinal activity. The drug to be tested was then washed in for the first time (Drug 1st) and 20 min later the retinal activity under the influence of the drug was recorded before the drug was washed out again with Ames' medium after another 20 min (Ames' 2nd). Wash-in and wash-out of the drug were repeated (Drug 2nd, Ames' 3rd) to confirm the effect of the drug. Electrophysiological recordings included the spontaneous activity of retinal neurons and their response to square-wave pulses of ± 40-100 µA. **(b)** Overall view of the retinal electrical activity recorded from the 60 individual MEA channels using the Multi Channel Experimenter software

(light grey box: two example channels for the detailed view in (c); channel marked with #: reference electrode; channels marked with *: position of the optic nerve head). **(c)** *Rd10* retinal activity recorded in two adjacent channels using the Multi Channel Experimenter software, shown as raw data (top row), as 50 Hz low pass filtered data (middle row), and as 200-2000 Hz band-pass filtered data (bottom row; x-axis: 500 ms, y-axis: ± 100 µV). **(d)** Analysis of the raw data with the NeuroExplorer software to calculate the power spectral density including the fast Fourier transform to determine the main oscillation frequency [Hz] in the local field potential of each channel. **(e)** Evaluation of the raw data with the Offline Sorter software for spike sorting of a clearly distinguishable cell (marked in yellow). Display of the spike cutouts as overlay in the waveform window (top left), as 2D cluster with x- and y-axis as principal components 1 and 2 (top right) and over time (bottom; scale: 100 ms). **(f)** Analysis of sorted spikes using NeuroExplorer software to determine the spike rate of individual retinal neurons immediately before and after the application of an electrical stimulus (inverted triangle). As examples, a sequence of spikes in bursts (top row) and an increasing number of spikes immediately after the stimulus (bottom row) are shown.
